# Supplementary material for: Immune Checkpoint Inhibitor Use in Advanced Hepatocellular Carcinoma: A Real-World Analysis of Efficacy and Toxicity
Source: Cancers (Basel). 2025 Sep 17;17(18):3034. doi: 10.3390/cancers17183034 (PMC12468603; doi:10.3390/cancers17183034)
Supplement: Supplementary file 1 [file cancers-17-03034-s001.zip › cancers-3872255-supplementary.pdf]

Table S1. A comprehensive list of univariate analyses of factors influencing survival.

	**Overall survival (OS)**			**ICI-Specific Survival (OS-ICI)**			**Progression-Free Survival (PFS)**		
	HR	95% CI	*p*-value	HR	95% CI	*p*-value	HR	95% CI	*p*-value
Gender									
Female	-	-		-	-		-	-	
Male	1.86	0.84, 4.10	0.12	1.24	0.62, 2.51	0.5	1.63	0.81, 3.29	0.2
Race									
White	-	-		-	-		-	-	
African-American	1.11	0.49, 2.50	0.8	0.73	0.31, 1.73	0.5	0.59	0.25, 1.41	0.2
Other	0.76	0.10, 5.57	0.8	2.43	0.32, 18.3	0.4	2.38	0.32, 17.9	0.4
Age	0.98	0.95, 1.01	0.2	0.97	0.94, 1.00	0.060	0.98	0.95, 1.01	0.2
Alcohol history									
no	-	-		-	-		-	-	
yes	1.74	0.94, 3.22	0.077	2.07	1.11, 3.84	0.021	2.10	1.15, 3.83	0.016
Hepatitis C									
no	-	-		-	-		-	-	
Yes	1.06	0.60, 1.88	0.8	0.97	0.55, 1.72	>0.9	0.79	0.45, 1.40	0.4
Barcelona Clinic Liver Cancer									
B	-	-		-	-		-	-	
C	1.83	0.58, 5.81	0.3	1.39	0.49, 3.98	0.5	1.46	0.51, 4.18	0.5
D	2.12	0.74, 6.07	0.2	1.34	0.52, 3.46	0.5	1.39	0.54, 3.60	0.5
Line of immune-checkpoint inhibitor use									
1	-	-		-	-		-	-	
>1	0.67	0.36, 1.21	0.2	1.46	0.79, 2.69	0.2	1.74	0.96, 3.17	0.070
Systemic Therapy									
None	-	-		-	-		-	-	
lenvatinib (len.)	1.09	0.15, 8.13	>0.9	8.42	1.01, 70.3	0.049	7.08	0.87, 57.7	0.067
sorafenib (sor.)	1.18	0.62, 2.24	0.6	1.46	0.77, 2.75	0.2	1.96	1.01, 3.79	0.047
sor. + regorafenib	0.91	0.12, 6.79	>0.9	5.74	0.71, 46.1	0.10	5.18	0.65, 41.2	0.12
sor. + len.	1.43	0.19, 10.8	0.7	17.3	1.86, 161	0.012	12.1	1.40, 105	0.023
sor. + len + ramicurimab	1.36	0.18, 10.2	0.8	1.25	0.17, 9.32	0.8	1.00	0.13, 7.46	>0.9
Bevacizumab Combo									
no	-	-		-	-		-	-	
yes	0.62	0.31, 1.25	0.2	0.46	0.23, 0.93	0.032	0.59	0.31, 1.15	0.12
Ablation									
no	-	-		-	-		-	-	
yes	0.35	0.13, 0.90	0.029	0.69	0.28, 1.70	0.4	0.76	0.32, 1.84	0.5
Transarterial chemoembolization									
no	-	-		-	-		-	-	
yes	0.59	0.31, 1.12	0.11	0.90	0.48, 1.71	0.8	0.92	0.49, 1.71	0.8
Transarterial radioembolization									
no	-	-		-	-		-	-	
yes	0.36	0.17, 0.79	0.011	0.53	0.25, 1.15	0.11	0.70	0.35, 1.40	0.3
Surgery									
no	-	-		-	-		-	-	
yes	0.52	0.20, 1.37	0.2	0.72	0.27, 1.88	0.5	0.94	0.36, 2.45	>0.9
Any previous locoregional therapy (LRT)									
none	-	-		-	-		-	-	
yes	0.53	0.29, 0.95	0.033	1.09	0.61, 1.94	0.8	1.28	0.73, 2.24	0.4

Any previous systemic and locoregional therapy									
no	-	-		-	-		-	-	
yes	0.67	0.36, 1.21	0.2	1.46	0.79, 2.69	0.2	1.74	0.96, 3.17	0.070
Size 3 cm									
≤3	-	-		-	-		-	-	
>3	1.18	0.59, 2.39	0.6	0.50	0.25, 1.00	0.051	0.69	0.35, 1.36	0.3
Stereotactic body radiation therapy									
no	-	-		-	-		-	-	
yes	1.08	0.54, 2.19	0.8	1.00	0.50, 2.02	>0.9	1.29	0.63, 2.62	0.5
White blood cell count	0.99	0.89, 1.11	0.9	0.95	0.85, 1.07	0.4	0.97	0.88, 1.08	0.6
Red blood cell count	1.0	0.65, 1.52	>0.9	0.78	0.50, 1.22	0.3	0.66	0.42, 1.05	0.076
Hemoglobin	0.94	0.83, 1.08	0.4	0.87	0.76, 1.0	0.042	0.84	0.73, 0.96	0.013
Platelets	1.00	1.00, 1.00	0.5	1.00	1.00, 1.00	0.6	1.00	1.00, 1.00	0.7
Absolute neutrophil count	1.03	0.90, 1.17	0.7	0.99	0.87, 1.13	>0.9	1.00	0.88, 1.13	>0.9
Absolute lymphocyte count	0.97	0.72, 1.32	0.9	0.76	0.53, 1.09	0.13	0.83	0.59, 1.17	0.3
Total bilirubin	0.98	0.78, 1.23	0.9	1.17	0.89, 1.54	0.3	1.31	0.96, 1.78	0.085
Albumin	0.69	0.42, 1.12	0.14	0.60	0.36, 0.98	0.042	0.53	0.32, 0.87	0.013
Platelet-lymphocyte ratio	1.00	1.00, 1.00	0.7	1.00	1.00, 1.01	0.049	1.00	1.00, 1.01	0.045
Neutrophil to lymphocyte ratio	0.98	0.92, 1.03	0.4	1.04	0.98, 1.10	0.2	1.08	1.01, 1.15	0.033
Albumin bilirubin (ALBI) Score									
1	-	-		-	-		-	-	
2	2.33	0.96, 5.64	0.061	1.69	0.72, 3.97	0.2	2.21	0.89, 5.52	0.089
3	2.05	0.80, 5.20	0.13	2.35	0.91, 6.03	0.076	3.51	1.28, 9.63	0.015
Alkaline phosphatase	1.00	1.00, 1.00	0.3	1.00	1.00, 1.00	0.11	1.00	1.00, 1.00	0.4
Alanine aminotransferase	1.00	1.00, 1.00	0.3	1.00	1.00, 1.00	0.11	1.00	1.00, 1.00	0.4
Aspartate aminotransferase	1.00	1.00, 1.01	0.6	1.00	0.99, 1.01	0.8	1.00	1.00, 1.01	0.4
Thyroid stimulating hormone	0.94	0.86, 1.03	0.2	0.99	0.92, 1.07	0.8	0.97	0.89, 1.05	0.5
T4	0.36	0.05, 2.45	0.3	0.67	0.11, 4.17	0.7	0.34	0.05, 2.45	0.3
Ascites									
no	-	-		-	-		-	-	
yes	1.28	0.70, 2.32	0.4	1.01	0.56, 1.82	>0.9	1.00	0.56, 1.80	>0.9
Encephalopathy									
no	-	-		-	-		-	-	
yes	0.99	0.47, 2.05	>0.9	0.86	0.41, 1.78	0.7	0.72	0.36, 1.44	0.3
Child-Pugh Turcotte Score									
A5	-	-		-	-		-	-	
A6	1.60	0.72, 3.54	0.3	2.22	0.96, 5.11	0.062	2.00	0.88, 4.54	0.10
B7	0.91	0.26, 3.24	0.9	0.68	0.19, 2.42	0.6	0.64	0.21, 1.97	0.4
B8	1.44	0.50, 4.12	0.5	1.59	0.56, 4.57	0.4	1.51	0.52, 4.39	0.4
B9	1.82	0.74, 4.50	0.2	1.64	0.66, 4.08	0.3	1.97	0.80, 4.88	0.14
C10	1.40	0.49, 4.02	0.5	7.24	2.33, 22.5	<0.001	9.25	2.88, 29.7	<0.001
C11	4.74	0.58, 38.8	0.15	2.61	0.32, 21.0	0.4	2.27	0.28, 18.2	0.4
Child-Pugh Turcotte Category									
A	-	-		-	-		-	-	
B	1.16	0.62, 2.17	0.7	0.92	0.49, 1.73	0.8	0.94	0.51, 1.72	0.8
C	1.35	0.54, 3.36	0.5	3.64	1.42, 9.33	0.007	4.99	1.91, 13.1	0.001

Immune-related adverse events									
no	-	-		-	-		-	-	
yes	2.00	0.97, 4.10	0.059	2.01	0.97, 4.15	0.059	1.45	0.72, 2.92	0.3

HR–hazard ratio, CI–confidence interval.

Table S2. Mean change in the blood count parameters associated with progression.

**Parameter**	**Baseline (*n* = 43)**[1]	**After ICI (*n* = 43)**[1]	**Difference**[2]	**95% CI**[2,3]	***p*-value**[2]
WBC	6.7 (3.0)	7.1 (3.1)	-0.384	-1.54, 0.773	0.5
RBC	3.97 (0.78)	3.94 (0.65)	0.032	-0.153, 0.218	0.7
Hb	11.93 (2.31)	11.73 (2.19)	0.205	-0.332, 0.741	0.4
Platelets	196 (125)	170 (74)	26.3	-7.81, 60.3	0.13
ANC	4.64 (2.56)	4.86 (2.83)	-0.221	-1.23, 0.785	0.7
Lymphocytes	1.28 (0.91)	0.99 (0.54)	0.295	0.063, 0.527	0.014

[1] Mean (SD), [2] Paired t-test, [3] CI = Confidence Interval.

Table S3. Baseline characteristics of the cohort stratified by irAE status.

**Variable**	**No irAE (*n* = 43)**	**irAE (*n* = 10)**	***P* value**
Age	66 (8)	67 (13)	0.7
Gender			>0.9
Female	8 (19%)	2 (20%)	
Male	35 (81%)	8 (80%)	
Race			>0.9
White	36 (84%)	9 (90%)	
African-American	6 (14%)	1 (10%)	
Other	1 (2.3%)	0 (0%)	
Hepatitis C	19 (44%)	5 (50%)	>0.9
Alcohol History	28 (65%)	7 (70%)	>0.9
Barcelona Clinic Liver Cancer Stage			0.2
B	3 (7.0%)	2 (20%)	
C	9 (21%)	3 (30%)	
D	31 (72%)	5 (50%)	
Line of immune-checkpoint inhibitor use			0.5
1	15 (35%)	5 (50%)	
>1	28 (65%)	5 (50%)	
Systemic Therapy			0.3
None	26 (60%)	8 (80%)	
lenvatinib (len.)	1 (2.3%)	0 (0%)	
sorafenib (sor.)	14 (33%)	1 (10%)	
sor. + regorafenib	1 (2.3%)	0 (0%)	
sor. + len.	1 (2.3%)	0 (0%)	
sor. + len. + ramicurimab	0 (0%)	1 (10%)	
Bevacizumab combination	13 (30%)	0 (0%)	0.10
Size 3 cm			0.4
≤3	8 (19%)	3 (30%)	
>3	35 (81%)	7 (70%)	
Ablation	6 (14%)	0 (0%)	0.6
TACE	11 (26%)	3 (30%)	>0.9
TACE	7 (16%)	3 (30%)	0.4
SBRT	9 (21%)	1 (10%)	0.7
Surgery	5 (12%)	0 (0%)	0.6

Any previous LRT			0.7
None	21 (49%)	6 (60%)	
Yes	22 (51%)	4 (40%)	
Previous systemic and LRT	28 (65%)	5 (50%)	0.5
Total bilirubin	1.54 (1.11)	0.99 (0.46)	0.2
Albumin	3.26 (0.62)	3.40 (0.39)	0.5
Albumin – bilirubin score			0.4
1	7 (16%)	1 (10%)	
2	23 (53%)	8 (80%)	
3	13 (30%)	1 (10%)	
PLR	179 (97)	203 (147)	>0.9
NLR	5.4 (5.1)	3.5 (2.0)	0.3
ALP	221 (154)	165 (86)	0.3
ALT	221 (154)	165 (86)	0.3
AST	82 (50)	59 (25)	0.2
TSH	3.58 (3.27)	4.03 (3.18)	0.5
T4	1.10 (0.15)	1.15 (0.16)	0.4
Ascites	31 (72%)	4 (40%)	0.071
Encephalopathy	9 (21%)	2 (20%)	> 0.9
Child – Pugh score			0.2
A5	11 (26%)	3 (30%)	
A6	9 (21%)	5 (50%)	
B7	5 (12%)	0 (0%)	
B8	3 (7.0%)	2 (20%)	
B9	9 (21%)	0 (0%)	
C10	5 (12%)	0 (0%)	
C11	1 (2.3%)	0 (0%)	
Child-Pugh Category			0.2
CP A	20 (47%)	8 (80%)	
CP B	17 (40%)	2 (20%)	
CP C	6 (14%)	0 (0%)	
WBC	6.63 (3.10)	5.53 (1.68)	0.5
RBC	3.88 (0.69)	4.37 (0.87)	0.13
Hemoglobin	11.86 (2.34)	12.56 (1.14)	0.3
Platelets	181 (115)	206 (132)	0.4
ANC	4.58 (2.64)	3.39 (0.88)	0.3
Lymphocyte count	1.25 (0.87)	1.31 (0.87)	>0.9
WBC_2	7.31 (3.26)	5.75 (0.65)	0.2
RBC_2	3.81 (0.67)	4.35 (0.55)	0.050
Hb_2	11.63 (2.42)	11.95 (0.93)	0.8
Platelets_2	168 (75)	178 (73)	>0.9
ANC_2	5.10 (3.04)	3.58 (0.24)	0.3
Lymphocytes count_2	1.01 (0.58)	0.85 (0.21)	0.7

2 – at the time of immune-related adverse event, TACE - transarterial chemoembolization, TARE - transarterial radioembolization (TARE), SBRT - stereotactic body radiation therapy, LRT – locoregional therapy, NLR – neutrophil to lymphocyte ratio, PLR - platelet to lymphocyte ratio, ALP - albumin, alkaline phosphatase, ALT - alanine transferase. AST - aspartate transferase, WBC - white blood cells, RBC - red blood cells , ANC - absolute neutrophilic count,.

Table S4. Predictive Factors of irAE in Univariate and Multivariate Analysis.

		Univariable (irAE)			Multivariable (irAE)		
Characteristic	N	OR[1]	95% CI[1]	*P*-value	OR[1]	95% CI[1]	*P*-value
AST	53	0.98	0.96, 1.00	0.2			
Ascites	53						
No		-	-		-	-	
Yes		0.26	0.06, 1.06	0.063	0.26	0.06, 1.06	0.063
CP category	53						
CP A		-	-				
CP B		0.29	0.04, 1.37	0.2			
CP C		0.00		>0.9			

[1] OR = Odds Ratio, CI = Confidence Interval.

Table S5. Mean change in the blood count from baseline to irAE occurrence.

**Blood count**	**Baseline (*n* = 8)[1]**	**At irAE occurrence (*n* = 8)[1]**	**Differ-ence[2]**	**95% CI[2,3]**	***P*–value[2]**
WBC	5.69 (1.60)	5.75 (0.65)	-0.421	-1.22, 0.750	0.4
RBC	4.40 (0.97)	4.35 (0.55)	-0.046	-0.720, 0.660	>0.9
Hb	12.41 (1.24)	11.95 (0.93)	0.998	-0.700, 1.40	0.2
Platelets	220 (140)	178 (73)	44.0	-70.0, 112	0.5
ANC	3.65 (0.67)	3.58 (0.24)	0.069	-0.240, 0.310	>0.9
Lymphocytes	1.18 (0.81)	0.85 (0.21)	0.196	-0.280, 0.880	>0.9

[1] Mean (SD), [2] Wilcoxon rank sum test, [3] CI = Confidence Interval.

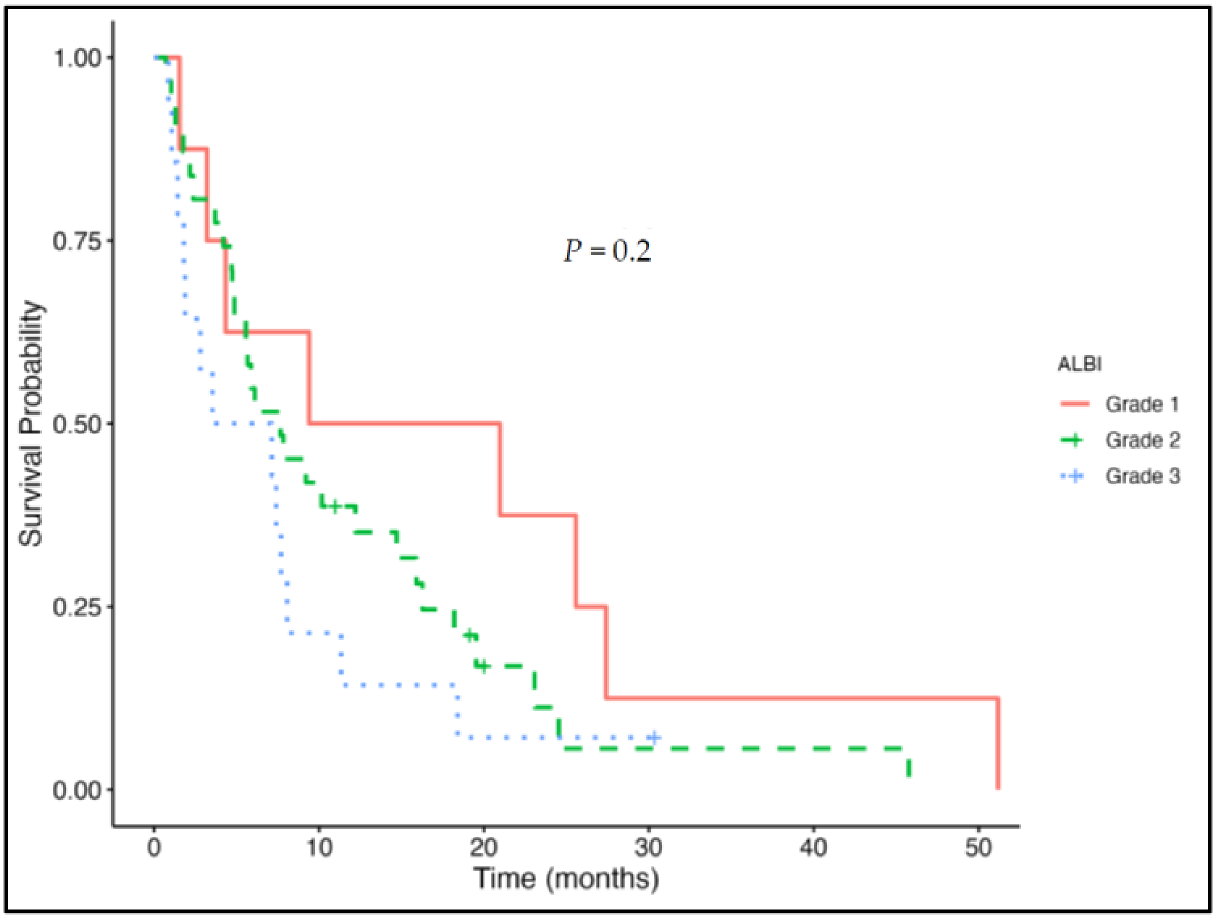

Figure S1. Kaplan-Meier Curve for ICI-specific overall survival (OS-ICI) by albumin – bilirubin (ALBI) Grade.

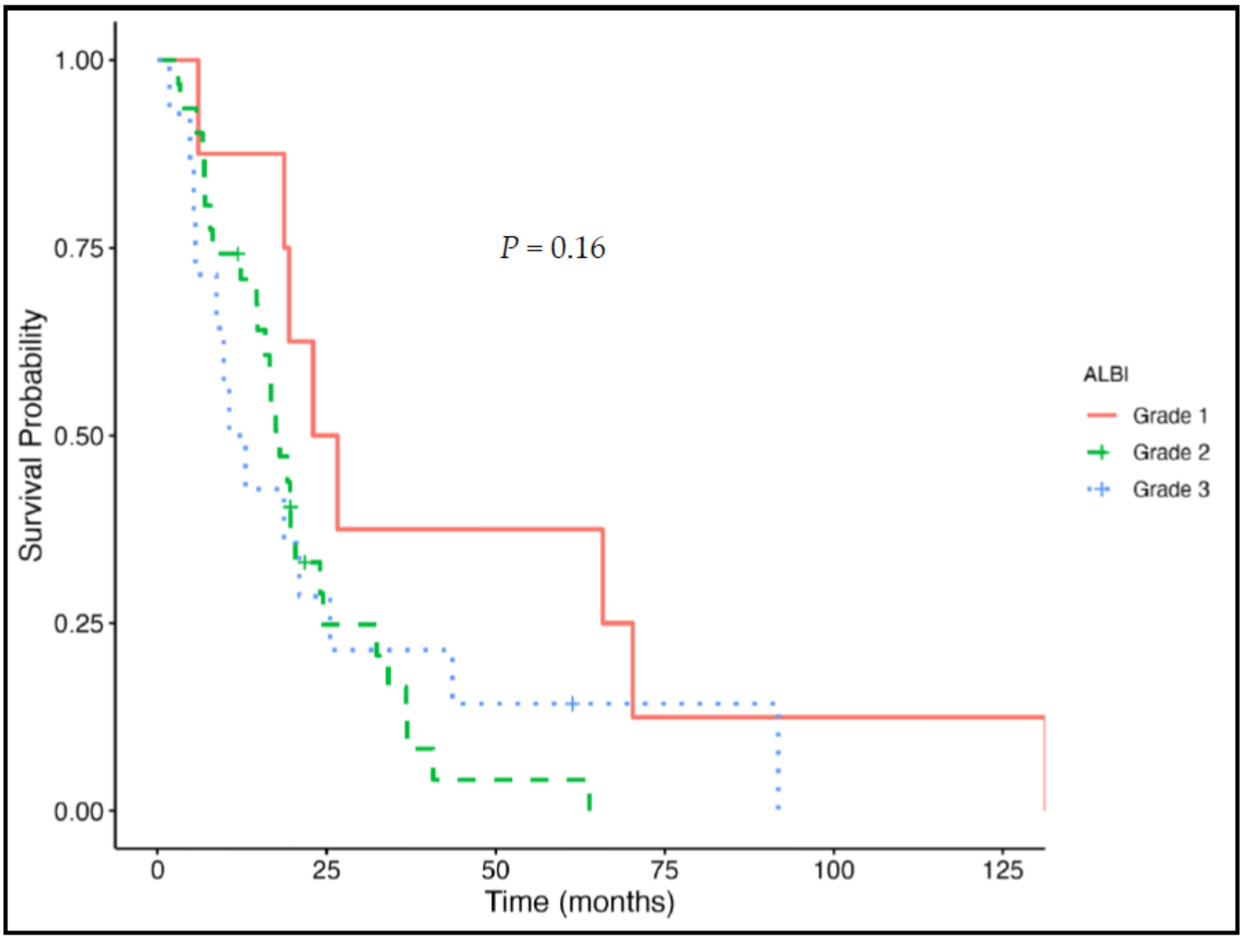

Figure S2. Kaplan-Meier Curve for overall survival by albumin – bilirubin (ALBI) Grade.
